# Posterior dislocation of the elbow associated with fracture of the radial head and olecranon, and with medial collateral ligament disruption: A case report

**DOI:** 10.1186/1757-1626-1-168

**Published:** 2008-09-19

**Authors:** Hua Chen, Peifu Tang, Boxun Zhang

**Affiliations:** 1 Department of Orthopaedics and Trauma Surgery, PLA General Hospital, Beijing, PR China

## Abstract

**Introduction:**

Fracture dislocations of the elbow appear extremely complex. Identification of the basic injury patterns can facilitate management.

**Case presentation:**

A 38-year-old male motor-vehicle driver who fell on his right elbow after an accident was suffering from posterior dislocation of the elbow, without coronoid fracture, and with fracture of the radial head and olecranon, and medial collateral ligament disruption, which was not associated with any vascular or neural injury.

**Conclusion:**

Posterior dislocation of the elbow associated with fracture of the radial head and olecranon, and medial collateral ligament disruption may be a rare subgroup of elbow dislocation. We should pay more attention to medial collateral ligament injury with elbow dislocation.

## Introduction

Fracture dislocations of the elbow appear extremely complex. Identification of the basic injury patterns can facilitate management. The simplest pattern of elbow fracture dislocation is posterior dislocation of the elbow with fracture of the radial head. Addition of a coronoid fracture, no matter how small, to elbow dislocation and radial head fracture is called the "terrible triad of the elbow" [1,2]. However, the present case highlights addition of an olecranon fracture to posterior dislocation of the elbow and radial head fracture. Medial collateral ligaments were also completely disrupted.

## Case presentation

**Figure 1:**
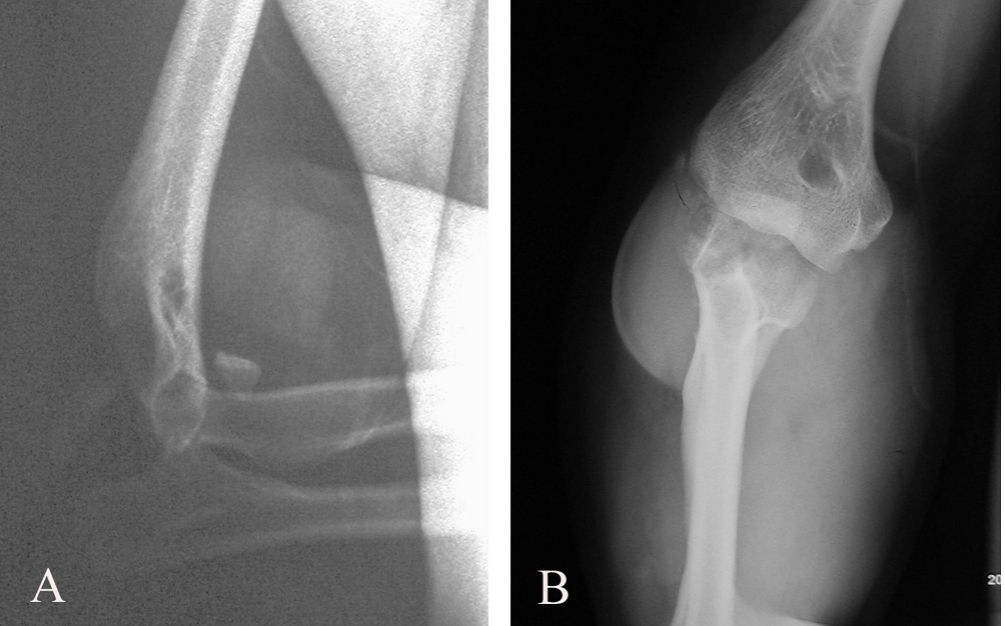
X-ray images of posterior elbow dislocation with fracture of the olecranon and radial head.

A 38-year-old male motor-vehicle driver presented to the emergency department after falling on his right elbow and dislocating his elbow. On admission, there was a palpable radial pulse and full sensation in his forearm and hand. Radiography revealed this to be a posterior elbow dislocation, with fracture of the olecranon and radial head (Figure 1A and 1B).

**Figure 2:**
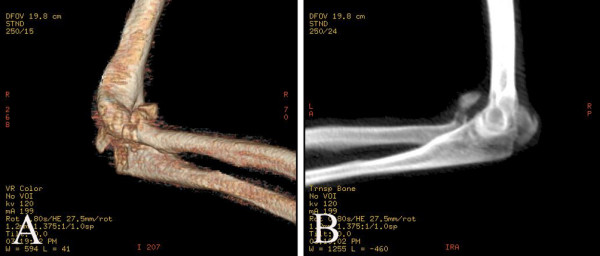
3D CT of posterior elbow dislocation with fracture of the olecranon and radial head, without coronoid fracture.

**Figure 3:**
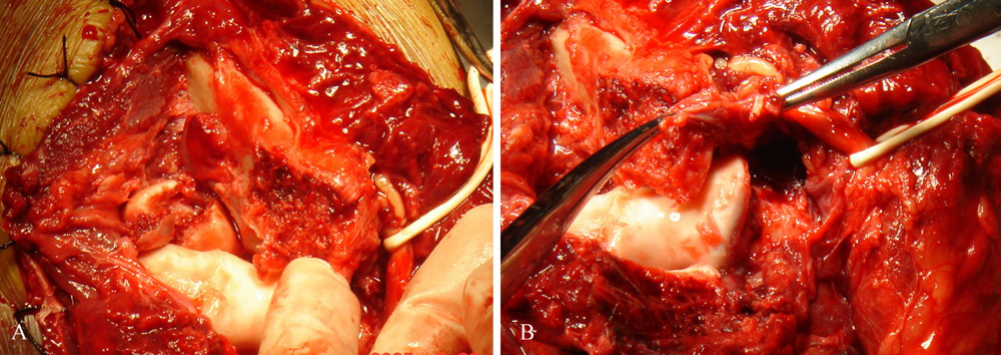
Olecranon simple oblique fracture, radial head comminuted fracture, medial collateral ligament disruption from the point of insertion to the distal humerus, which was not associated with neural injury.

The elbow was reduced immediately and fixed with plaster. Computer tomography and 3D reconstruction suggested posterior fracture dislocation of the elbow, with olecranon oblique fracture and radial head comminuted fracture, without coronoid fracture (Figure 2A and 2B). Surgery was carried out 6 days after injury. Posterior incision exposed the articular joint. Olecranon simple oblique fracture, radial head comminuted fracture and ulnar nerves were all visualized (Figure 3A).

**Figure 4:**
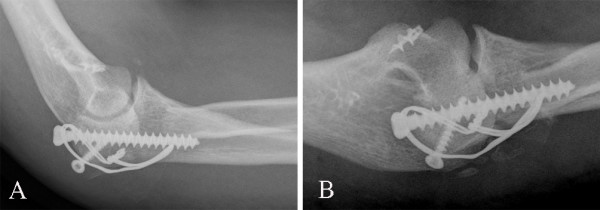
Medial collateral ligaments were repaired with a suture anchor, the radial head was resected, and the olecranon fracture was fixed with a lag screw and a tension band wire.

We found that medial collateral ligaments were completely disrupted from the point of insertion to the distal humerus (Figure 3B). Medial collateral ligaments were attached to the distal humerus with a 5-mm Fastin suture anchor (Depuy Mitek) (Figure 4A). The comminuted radial head fracture was resected. The olecranon fracture was fixed with a lag screw, 12 cm in length and 7.5 mm in diameter, and a tension band wire (Figure 4B). The elbow was placed in a back slab. The postoperative period was uneventful. Gentle physiotherapy was started after the elbow was maintained in a splint for 2 weeks. Functional recovery was complete in about 3 months. Six months after surgery, the active range of motion was 0/10/130 degrees of extension and flexion. Pronation and supination were full. There were no signs of elbow instability.

## Discussion

**Figure 5:**
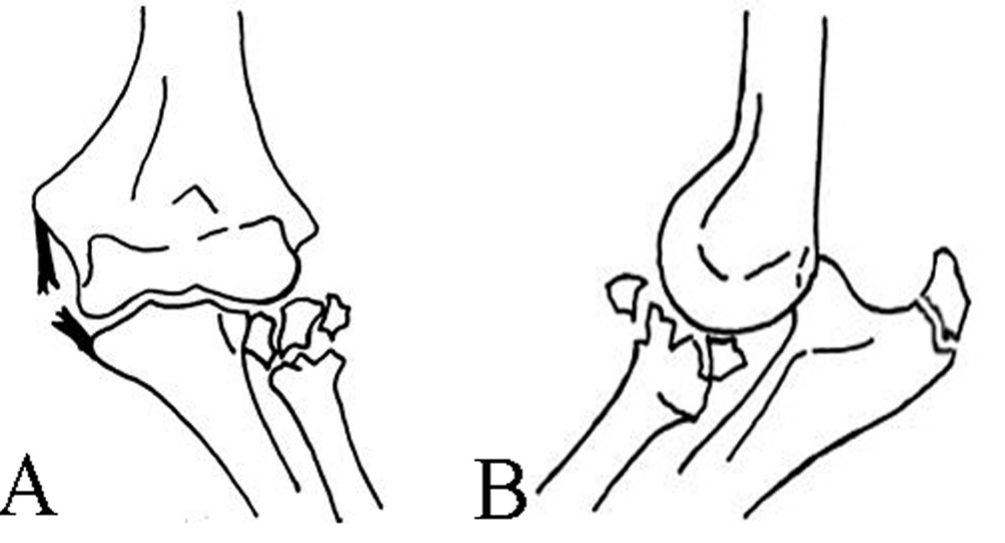
A new injury pattern associated with olecranon fracture dislocation without coronoid fractures, with radial head fracture and medial collateral ligament disruption, caused instability of the elbow.

Our case (Figure 5A and 5B) was different from those reported in the literature [3-9]. It may represent a rare subgroup [2]: posterior dislocation of the elbow associated with radial head fracture. It was associated with olecranon fracture and medial collateral ligament disruption. It was not the terrible-triad of posterior dislocation of the elbow with fracture of the radial head and coronoid process, because 3D scans confirmed that there was not any coronoid process fracture. It was not dislocation through the olecranon, in which the relationship of the humeroulnar joint is grossly retained, but the entire proximal forearm unit was dislocated with obvious radiocapitellar dislocation. It was not anterior trans-olecranon fracture-dislocation or posterior olecranon fracture-dislocation. The mechanism of injury might have been caused by falling onto the outstretched hand, with the elbow in extension and the arm in abduction in high energy injury.

We resected the radial head and restored the medial column with a suture anchor, and fixed the olecranon with a tension band and lag screws. The patient received early functional treatment and obtained a good outcome. In type 3 fractures [2], the radial head is replaced with a modular implant. The emphasis is placed on restoring the height of the radial head. Since Morrey and colleagues published their cadaver studies [10,11], the anterior band of the medial collateral ligament has been considered one of the most important stabilizers of the elbow. Their data suggest that the medial collateral ligament is the primary stabilizer of the elbow under valgus stress, with the radial head playing a less important, secondary role. According to clinical experience, it may be more appropriate to consider the functions of the medial collateral ligament and radial head as overlapping and complementary rather than hierarchical. A person with either an attenuated medial collateral ligament or an absent radial head can have difficulty performing activities that place a vigorous valgus stress on the elbow, such as throwing, but usually has little difficulty with normal daily activities. On the other hand, when the medial collateral ligament and radial head are injured simultaneously, the elbow can become very unstable and prone to subluxation or dislocation.

Therefore, it is more important to repair the medial collateral ligament for keeping the stability of the elbow than to restore the height of radial head. Accelerated functional treatment for elbow dislocation is important, as long periods of immobilization are not likely to be of any benefit.

## Conclusion

Posterior dislocation of the elbow associated with fracture of the radial head and olecranon, and medial collateral ligament disruption may be a rare subgroup of elbow dislocation. We should pay more attention to medial collateral ligament injury in elbow dislocation.

## Competing interests

The authors declare that they have no competing interests.

## Authors' contributions

CH, TPF and ZBX conducted the surgery, collected data and wrote the case report. ZBX planned and guided the surgery. All authors read and approved the final manuscript.

## Consent

Written informed consent was obtained from the patient for publication of the report and any accompanying images.
